# Supplementary figures and images for: “Paraxenoviridae”, a putative family of globally distributed marine bacteriophages with double-stranded RNA genomes
Source: ISME J. 2025 Jul 4;19(1):wraf139. doi: 10.1093/ismejo/wraf139 (PMC12445693; doi:10.1093/ismejo/wraf139)

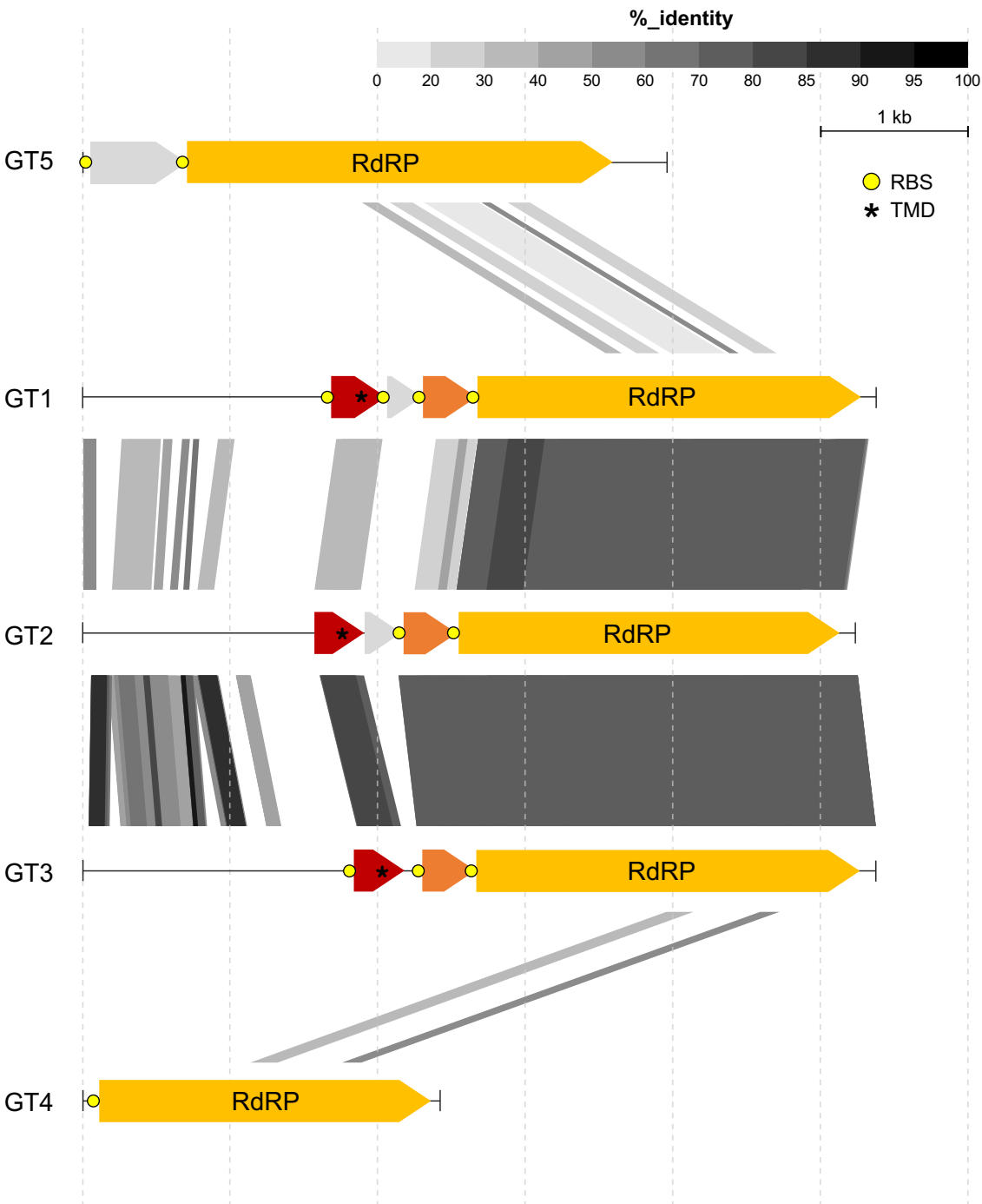

Supplement: 20250508_FigureS1_wraf139 [file 20250508_figures1_wraf139.pdf]

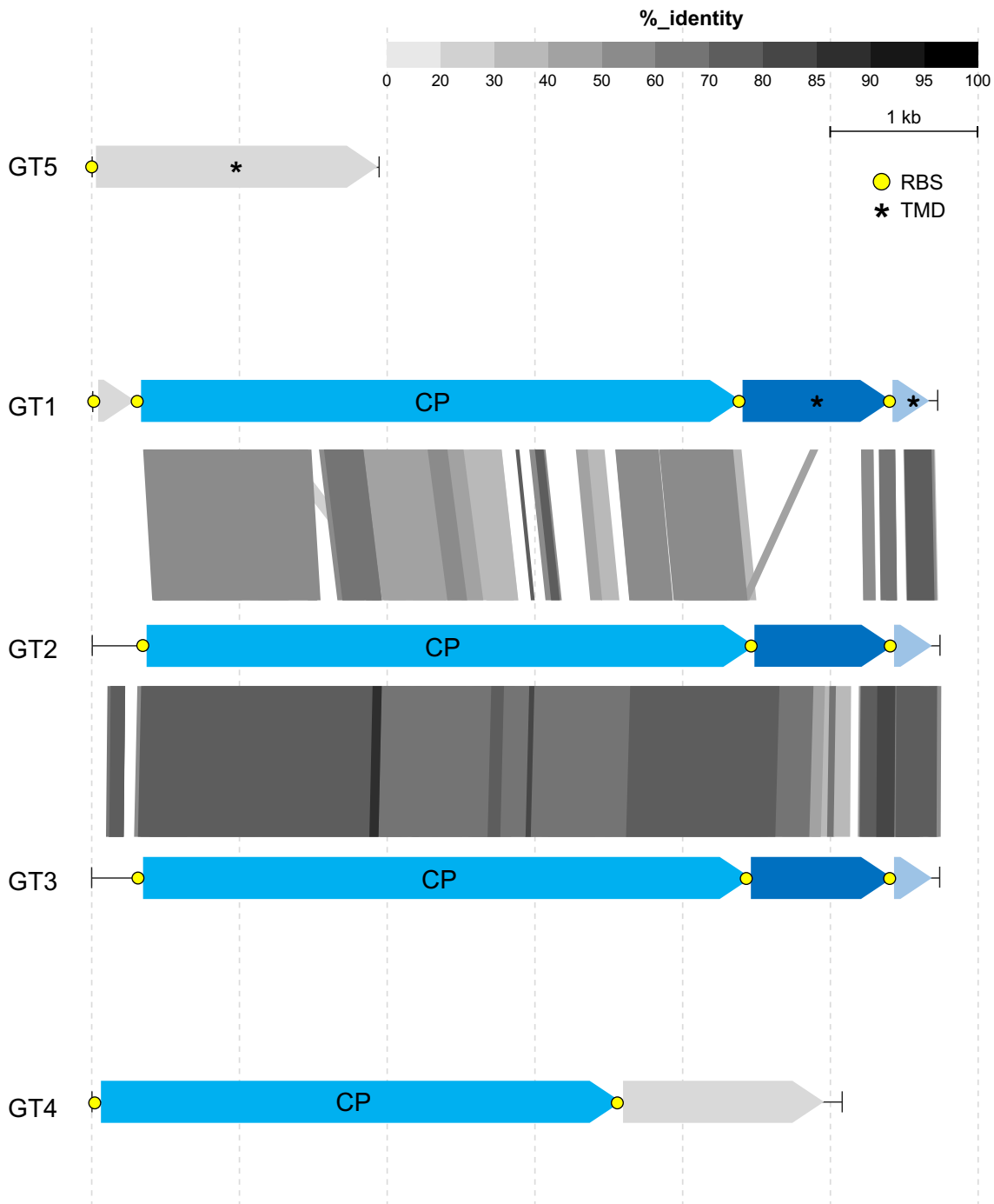

Supplement: 20250508_FigureS2_wraf139 [file 20250508_figures2_wraf139.pdf]

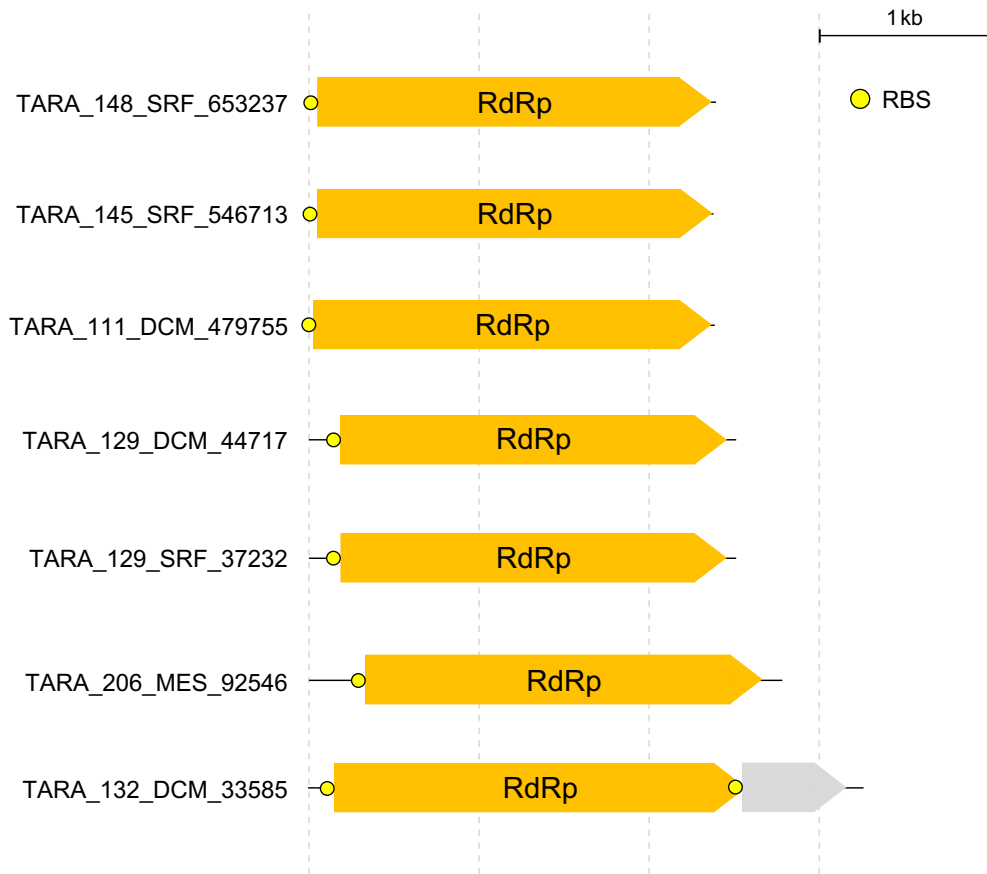

Supplement: 20250508_FigureS3_wraf139 [file 20250508_figures3_wraf139.pdf]

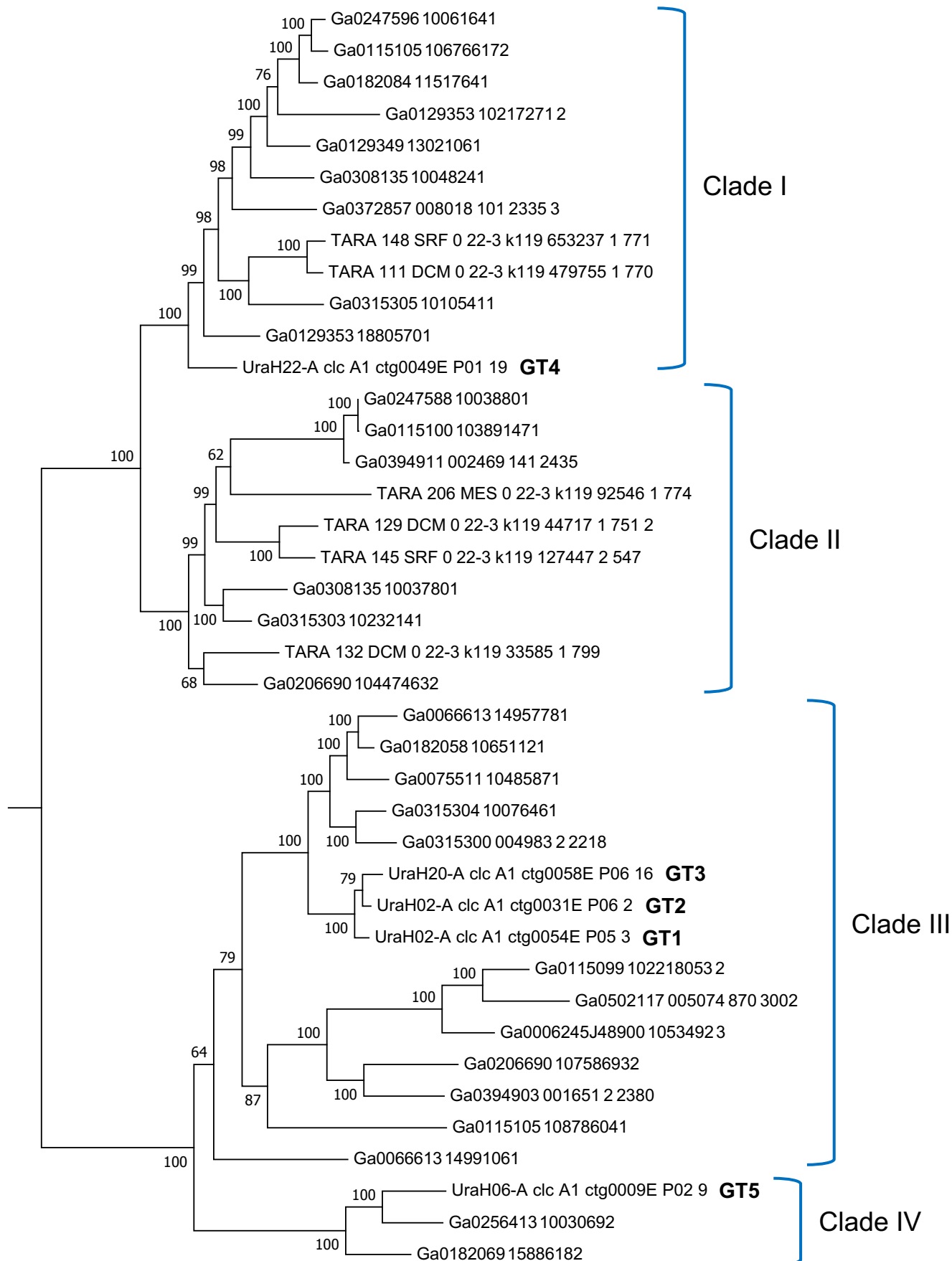

0.50

Supplement: 20250508_FigureS4_wraf139 [file 20250508_figures4_wraf139.pdf]

seed\_000 top AF2 model  
(Fig. 4A)

25 AF2 models superimposed  
(ptm\_models\_1~5 x seeds\_000~004)

TARA\_132

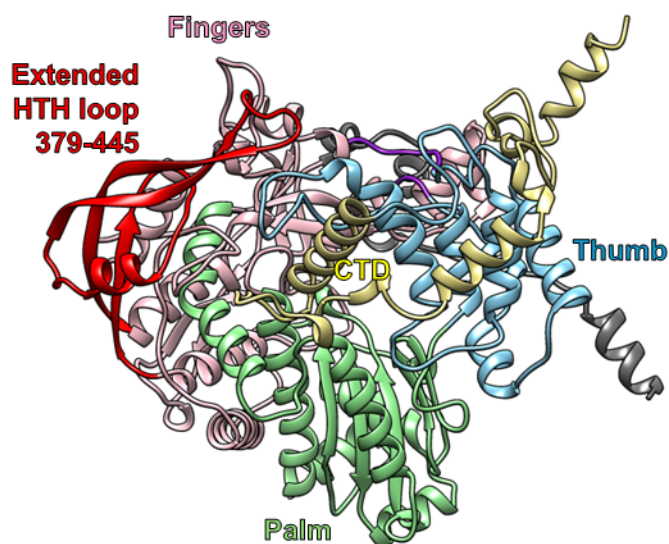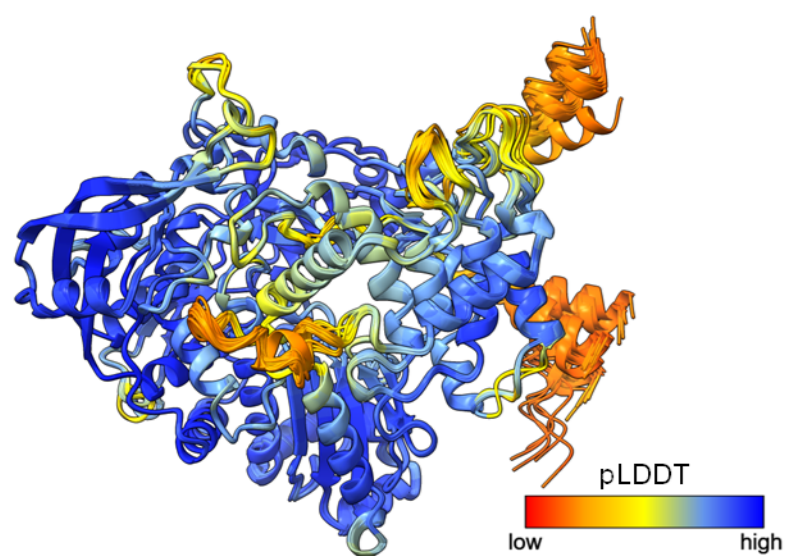

GT4

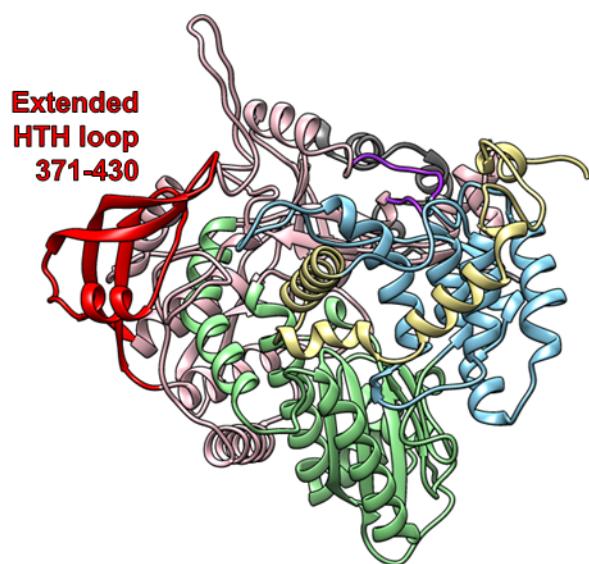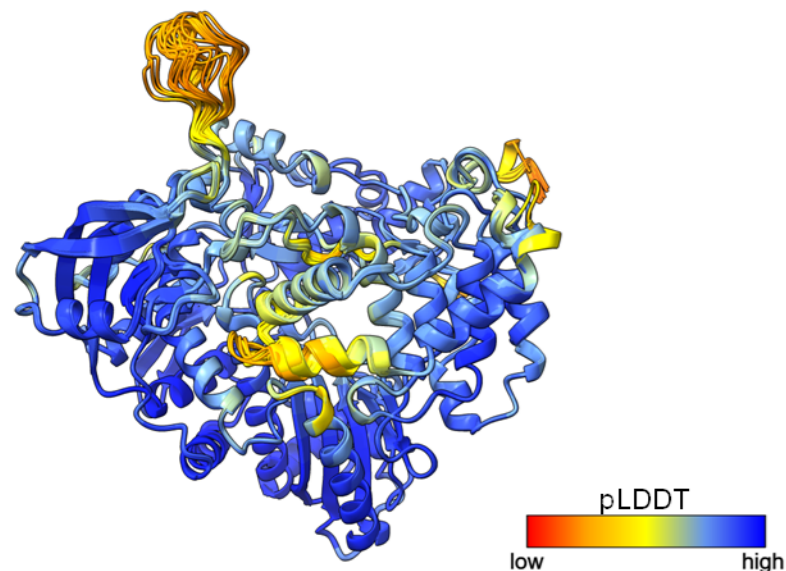

GT3

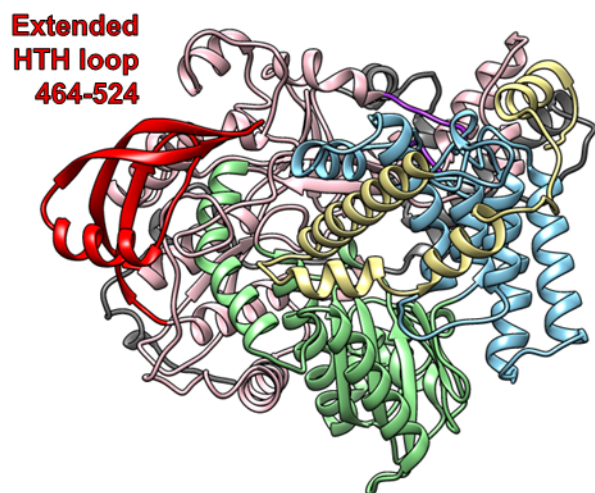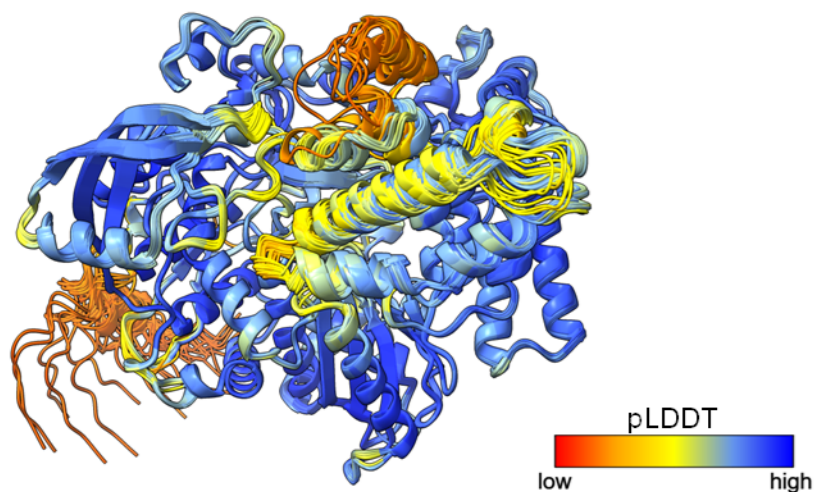

GT5

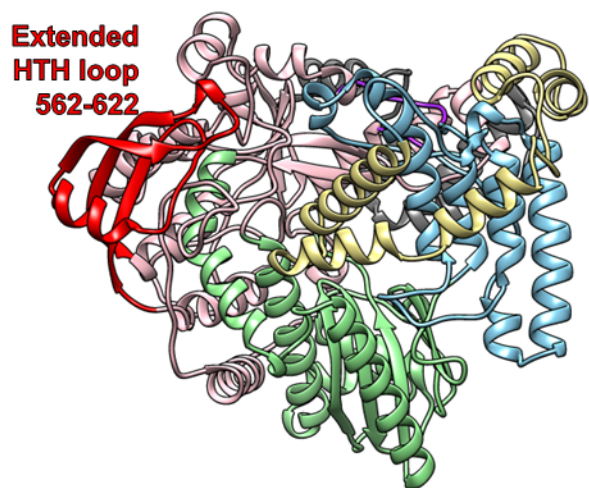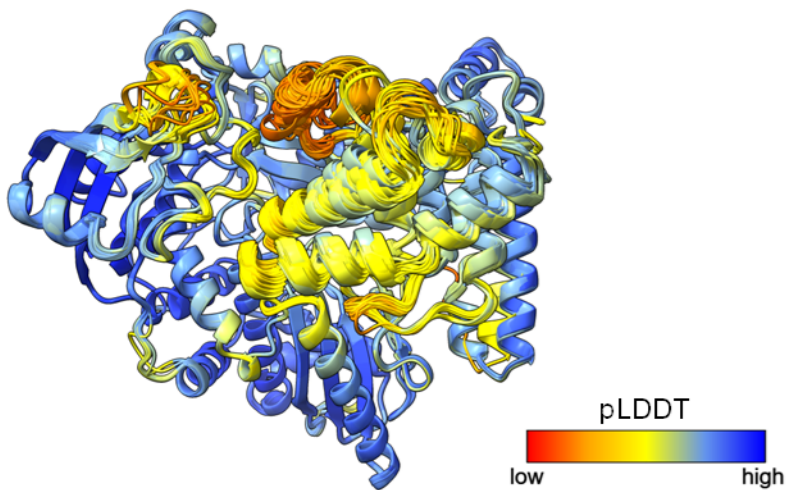

Supplement: 20250508_FigureS5_wraf139 [file 20250508_figures5_wraf139.pdf]

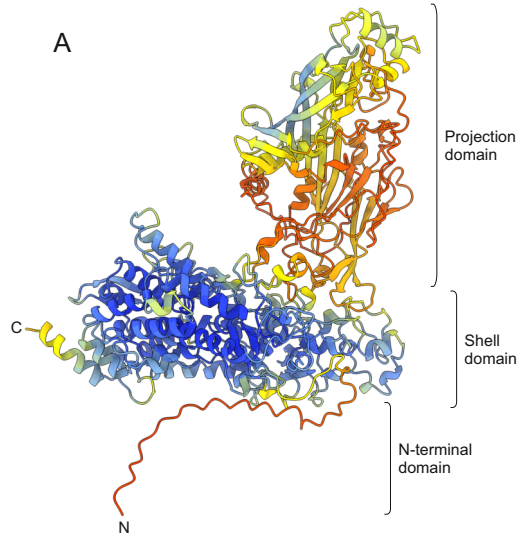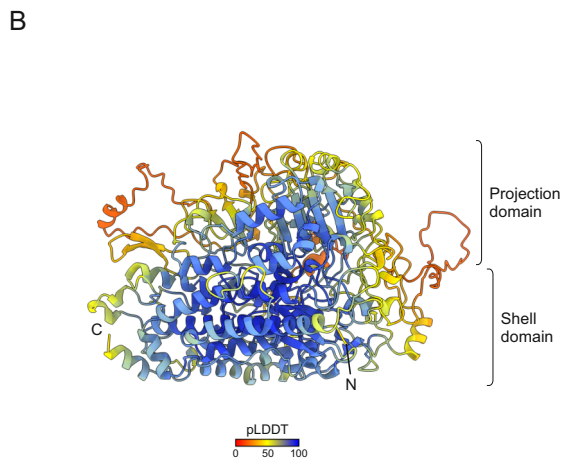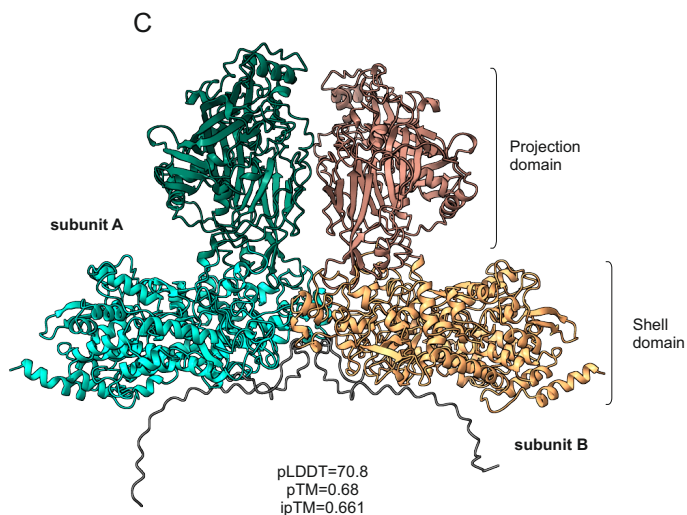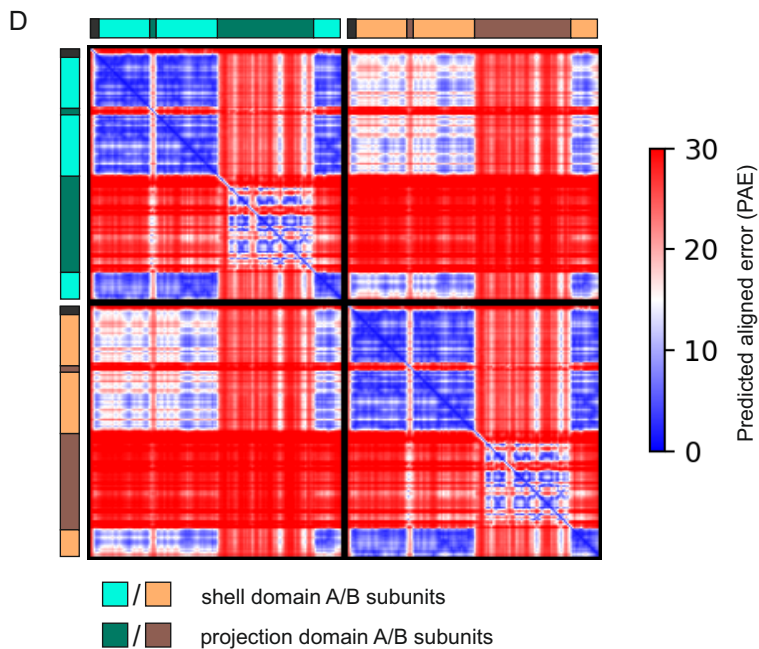

Supplement: 20250508_FigureS6_wraf139 [file 20250508_figures6_wraf139.pdf]

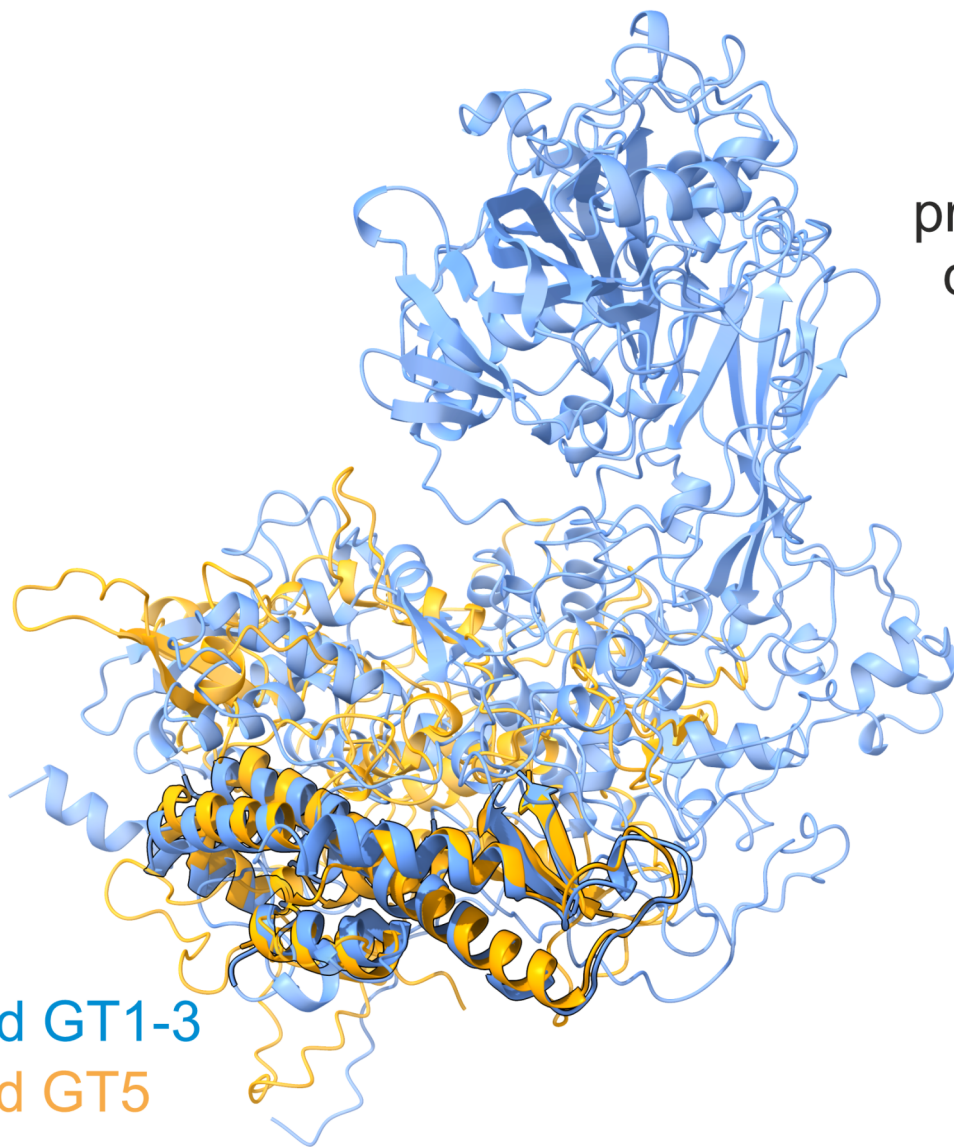

projection  
domain

shell  
domain

Capsid GT1-3

Capsid GT5

Supplement: 20250508_FigureS7_wraf139 [file 20250508_figures7_wraf139.pdf]
