# Supplementary material for: “Paraxenoviridae”, a putative family of globally distributed marine bacteriophages with double-stranded RNA genomes
Source: ISME J. 2025 Jul 4;19(1):wraf139. doi: 10.1093/ismejo/wraf139 (PMC12445693; doi:10.1093/ismejo/wraf139)
Supplement: 20250508_TableS1_wraf139 [file 20250508_tables1_wraf139.pdf]

Table S1. Overview of reads and contigs of the pelagic FLDS-drived virome libraries analyzed in this study.

Library name	Cleaned-up reads	Number of contigs	Total contig length (Mb)	N50 length (bp)	Mapped reads (%)	Taxonomic composition of mapped reads (%)			
						Viral contigs	Eukaryotic contigs	Bacterial contigs	Unassigned contigs
UraH2	1,611,256	1,917	1.83	974	46	6.8	3.3	1.0	88.9
UraH6	1,319,778	218	0.19	863	27	5.7	3.6	3.0	87.8
UraH20	2,980,518	1,673	1.50	892	50	15.1	1.4	0.8	82.7
UraH22	399,002	414	0.36	903	25	5.4	2.5	0.3	91.8
