# Supplementary material for: “Paraxenoviridae”, a putative family of globally distributed marine bacteriophages with double-stranded RNA genomes
Source: ISME J. 2025 Jul 4;19(1):wraf139. doi: 10.1093/ismejo/wraf139 (PMC12445693; doi:10.1093/ismejo/wraf139)
Supplement: 20250508_TableS2_wraf139 [file 20250508_tables2_wraf139.pdf]

Table S2. Summary of AlphaFold2 confidence scores for paraxenovirus RdRP predictions

RdRP	ptm_model	Seed	pLDDT	PTM	Rank
TARA_132	1	3	87.7	0.885	13
	1	1	87.5	0.884	15
	1	0	87.5	0.884	16
	1	2	87.3	0.884	20
	1	4	87.3	0.885	21
	2	3	87.9	0.895	11
	2	0	87.8	0.894	12
	2	4	87.5	0.894	17
	2	1	87.2	0.894	22
	2	2	86.4	0.89	24
	3	0	89.5	0.895	1
	3	3	89.3	0.893	2
	3	2	89.3	0.893	3
	3	1	89.2	0.891	4
	3	4	89.1	0.891	5
	4	1	88.4	0.887	6
	4	4	88.2	0.886	7
	4	2	88.1	0.885	8
	4	0	88.1	0.886	9
	4	3	88.1	0.886	10
	5	2	87.6	0.886	14
	5	1	87.5	0.887	18
	5	4	87.4	0.886	19
	5	0	86.7	0.885	23
	5	3	86.4	0.882	25
GT4	1	1	90.8	0.909	12
	1	0	90.6	0.908	13
	1	2	90.6	0.907	14
	1	4	90.6	0.908	15
	1	3	90.5	0.907	19
	2	0	91.2	0.915	6
	2	4	91.2	0.914	7
	2	1	91.2	0.915	8
	2	2	91.1	0.914	9
	2	3	90.9	0.913	10
	3	1	92	0.917	1
	3	0	91.8	0.914	2
	3	2	91.8	0.915	3
	3	4	91.7	0.915	4
	3	3	91.7	0.914	5
	4	1	90.8	0.91	11

	4	0	90.6	0.907	16
	4	2	90.6	0.908	17
	4	3	90.5	0.907	18
	4	4	90.3	0.908	20
	5	4	89.6	0.903	21
	5	3	89.6	0.904	22
	5	0	89.6	0.903	23
	5	2	89.6	0.903	24
	5	1	89.5	0.904	25
GT3	1	3	89.5	0.878	7
	1	0	89.2	0.876	11
	1	4	88.8	0.875	13
	1	1	86.9	0.859	20
	1	2	86.1	0.862	25
	2	4	89.9	0.894	5
	2	1	89.8	0.893	6
	2	0	87.1	0.883	19
	2	3	86.8	0.883	21
	2	2	86.7	0.88	23
	3	3	91.2	0.903	1
	3	4	91.2	0.902	2
	3	0	91.1	0.902	3
	3	1	90.3	0.897	4
	3	2	88.8	0.893	14
	4	3	89.4	0.882	8
	4	4	89.3	0.882	9
	4	1	89.1	0.881	12
	4	2	87.8	0.876	17
	4	0	87.3	0.876	18
	5	3	89.2	0.881	10
	5	4	88.7	0.88	15
	5	1	88.6	0.88	16
	5	2	86.7	0.869	22
	5	0	86.1	0.867	24
GT5	1	3	78.6	0.802	7
	1	4	77.7	0.798	11
	1	1	77.4	0.765	14
	1	0	76.9	0.767	19
	1	2	76.4	0.762	25
	2	4	77.6	0.785	12
	2	1	77.4	0.784	15
	2	2	77.3	0.788	17
	2	0	77	0.787	18

2	3	76.8	0.784	21
3	4	81.3	0.821	1
3	3	81.2	0.82	2
3	1	80	0.791	3
3	0	80	0.79	4
3	2	79	0.788	5
4	4	78.8	0.801	6
4	3	78.1	0.779	8
4	1	78	0.77	9
4	2	77.9	0.768	10
4	0	77.5	0.766	13
5	2	77.3	0.772	16
5	4	76.8	0.768	20
5	1	76.5	0.767	22
5	3	76.5	0.766	23
5	0	76.5	0.768	24
