# Supplementary material for: “Paraxenoviridae”, a putative family of globally distributed marine bacteriophages with double-stranded RNA genomes
Source: ISME J. 2025 Jul 4;19(1):wraf139. doi: 10.1093/ismejo/wraf139 (PMC12445693; doi:10.1093/ismejo/wraf139)
Supplement: 20250523_TableS3_new_wraf139 [file 20250523_tables3_new_wraf139.pdf]

Table S3. The complete genomes of the proposed phylum "*Candidatus* Paraxenoviricota"-associated RNA virus populations identified in this study and the previously reported TARA relatives (RdRP-encoded contigs; [11])

Complete genome group (group of segments)	Segment/contig name	Accession no.	Length (bp)	Average coverage	5'-terminal consensus region	3'-terminal consensus region	HMM search-based top hit (public & global marine virome RdRp_DB[1])	E-value	Library name	Source area
GT1	RNA1	LC876750	5,392	818	GTTAAATCATA	ACCT	TARA_145_SRF_0.22-3_k119_546713	7.0E-20	UraH2	Izu-Ogasawara Trench, Northwest Pacific Ocean
	RNA2	LC876751	5,738	748	GTTAAATCATA	ACCT	no hit	-	UraH2	Izu-Ogasawara Trench, Northwest Pacific Ocean
GT2	RNA1	LC876752	5,250	329	GTTAAATCGTAG	ACCT	TARA_132_DCM_0.22-3_k119_33585	1.0E-17	UraH2	Izu-Ogasawara Trench, Northwest Pacific Ocean
	RNA2	LC876753	5,757	322	GTTAAATCGTAG	ACCT	no hit	-	UraH2	Izu-Ogasawara Trench, Northwest Pacific Ocean
GT3	RNA1	LC876754	5,387	1,052	GTTAAATC	ACCT	TARA_145_SRF_0.22-3_k119_546713	1.0E-15	UraH20	Northeast Pacific Ocean
	RNA2	LC876755	5,759	1,808	GTTAAATC	ACCT	no hit	-	UraH20	Northeast Pacific Ocean
GT4	RNA1	LC876756	2,421	65	ACGAAGG	ACCT	TARA_111_DCM_0.22-3_k119_479755	0	UraH22	Okinawa Trough, East China Sea
	RNA2	LC876757	5,095	68	ACGAAGG	ACCC	no hit	-	UraH22	Okinawa Trough, East China Sea
GT5	RNA1	LC876758	3,971	452	ACAAAGAAAC	AACG	no hit	-	UraH6	East Indian Ocean
	RNA2	LC876759	1,953	464	ACAAAGAAAC	AACG	no hit	-	UraH6	East Indian Ocean
	TARA_148_SRF_653237	-	2,371	-	-	-	-	-	-	Tara Ocean sampling: Northwest Atlantic Ocean, surface depth
	TARA_145_SRF_546713	-	2,366	-	-	-	-	-	-	Tara Ocean sampling: Northwest Atlantic Ocean, surface depth
	TARA_111_DCM_479755	-	2,365	-	-	-	-	-	-	Tara Ocean sampling: Southeast Pacific Ocean, deep chlorophyll maximum depth
	TARA_129_DCM_44717	-	2,500	-	-	-	-	-	-	Tara Ocean sampling: Central Pacific Ocean, deep chlorophyll maximum depth
	TARA_129_SRF_37232	-	2,499	-	-	-	-	-	-	Tara Ocean sampling: Central Pacific Ocean, surface depth
	TARA_206_MES_92546	-	2,768	-	-	-	-	-	-	Tara Ocean sampling: Arctic Ocean, mesopelagic depth
	TARA_132_DCM_33585	-	3,245	-	-	-	-	-	-	Tara Ocean sampling: North Pacific Ocean, deep chlorophyll maximum depth

[1] Database of all available virus RdRps from Tara Oceans and coastal ocean viromes and GenBank, made by Zayed et al. [11]
