# Supplementary material for: “Paraxenoviridae”, a putative family of globally distributed marine bacteriophages with double-stranded RNA genomes
Source: ISME J. 2025 Jul 4;19(1):wraf139. doi: 10.1093/ismejo/wraf139 (PMC12445693; doi:10.1093/ismejo/wraf139)
Supplement: 20250714_TableS4_corrected_wraf139 [file 20250714_tables4_corrected_wraf139.pdf]

Table S4. The genomic organization and property of the predicted ORFs from the proposed phylum "*Candidatus* Paraxenoviricota"-associated RNA virus populations identified in this study and the previously reported TARA relatives

Segment	Length of segment (bp)	ORF	Strand	5'-coordinate	3'-coordinate	Product	Amino acids	Translation	Hit ORF (HHblits[1])	E-value	RBS	RBS spacer
GT1-RNA1	5,392	ORF1	+	1,689	2,063		124	MNEAESAKDINTEELTTNAGEEE	n/a	-	GGAGG	7 bp
		ORF2	+	2,069	2,311		80	MSEDVYITVTADGRATLIMTVM	n/a	-	GAG	4 bp
		ORF3	+	2,313	2,678		121	MMGIMTLRATDRFDWTEQYRG	n/a	-	AGGAGG	9 bp
		ORF4	+	2,683	5,289	RdRP	868	MGSQSDRRETGTAWVNDYARG	TARA_132_DCM_0.22-3_k119_33585 ORF1	8.00E-70	AGG	9 bp
GT1-RNA2	5,738	ORF1	+	44	289		81	MTRAGNTGTKVRWFGHRGSPE	n/a	-	GGAG	13 bp
		ORF2	+	329	4,405	Capsid	1,358	MDKKTDRSLKNERPSAQQAGG	n/a	-	AGGA	12 bp
		ORF3	+	4,413	5,426		337	MVLGIIDQASNWVVDQAEKAE	n/a	-	AGG	12 bp
		ORF4	+	5,431	5,682		83	MWKSRQQQPDRADEDPSTERDS	n/a	-	GAG	9 bp
GT2-RNA1	5,250	ORF1	+	1,575	1,913		112	MDDEESEMKIDDNTTTAGVGFE	n/a	-	no	
		ORF2	+	1,916	2,167		83	MVNAWTANIAGNILVVEPSPGL	n/a	-	no	
		ORF3	+	2,169	2,531		120	MTVLIPQFDHTQLEFVTETVNGG	n/a	-	AGGAGG	8 bp
		ORF4	+	2,555	5,143	RdRP	862	MKDQGTEWTDDYQRGYVRKID	TARA_132_DCM_0.22-3_k119_33585 ORF1	5.70E-60	GGA	11 bp
GT2-RNA2	5,757	ORF1	+	370	4,500	Capsid	1,376	MKGKTNTSHAGGSKGAANDRF	n/a	-	AGG	9 bp
		ORF2	+	4,497	5,438		313	MIKIPGVIDSIVGGVQDVTDAISI	n/a	-	AGG	11 bp
		ORF3	+	5,448	5,702		85	MKGVKSSRKEICHLDGVTPCSE	n/a	-	AGGAG	14 bp
GT3-RNA1	5,387	ORF1	+	1,841	2,185		114	MTTDNEIVTDDDNTTTAGVGFE	n/a	-	AGG	7 bp
		ORF2	+	2,306	2,668		120	MTALIAMNDHSNVQFTTVTENG	n/a	-	GGAGG	9 bp
		ORF3	+	2,674	5,280	RdRP	868	MGSSDRLKSQGKEWVEDYQRG	TARA_132_DCM_0.22-3_k119_33585 ORF1	1.10E-60	GAGG	8 bp
GT3-RNA2	5,759	ORF1	+	350	4,480	Capsid	1,376	MNNKKTKPNAKAVDHRASTNI	n/a	-	AGGAGG	8 bp
		ORF2	+	4,477	5,439		320	MITIPGVIGKIFDGVEDVMGAVS	n/a	-	AGG	11 bp
		ORF3	+	5,449	5,706		85	MLKGAKLKSKEICHLDGVTPCS	n/a	-	AGGAG	14 bp
GT4-RNA1	2,421	ORF1	+	111	2,357	RdRP	748	MEREIVLQNLAKLVDLNKLKKII	TARA_111_DCM_0.22-3_k119_479755 ORF1	1.00E-124	GGAGG	8 bp
GT4-RNA2	5,095	ORF1	+	62	3,595		1,177	MPTRILSTTSKPAEPTSTLNGLLA	n/a	-	AGGAGG	9 bp
		ORF2	+	3,607	4,971		454	MPTLAELLDEEEEYRDRLQEANI	n/a	-	GAGG	9 bp
GT5-RNA1	3,971	ORF1	+	55	711		218	MSNDNKSPFSKEDTSMELAESES	n/a	-	AGG	10 bp
		ORF2	+	708	3,602	RdRP	964	MKGRVLYNYVHAVERGDEARI	TARA_132_DCM_0.22-3_k119_33585 ORF1	5.70E-11	GAGG	7 bp
GT5-RNA2	1,953	ORF1	+	32	1,948		638	MICRNAFLEFPLDERYSDTISGEV	n/a	-	AGGAG	14 bp
TARA_148_SRF_653237	2,371	ORF1	+	51	2,342	RdRP	763	MAFLGTLNMAGDEPISQNRGFV	n/a	-	3Base/5BMM	13-15 bp
TARA_145_SRF_546713	2,366	ORF1	+	51	2,342	RdRP	763	MAFLGTLNMAGDEPISQNRGFV	n/a	-	3Base/5BMM	13-15 bp
TARA_111_DCM_479755	2,365	ORF1	+	30	2,342	RdRP	770	MRDHFVPNAPPGHLNVGLDGA	n/a	-	GAGG	8 bp
TARA_129_DCM_44717	2,500	ORF1	+	189	2,444	RdRP	751	MRKLKEIKPFLPAKVNGLTIRSFT	n/a	-	GGAGG	7 bp
TARA_129_SRF_37232	2,499	ORF1	+	189	2,444	RdRP	751	MRKLKEIKPFLPAKVNGLTIRSFT	n/a	-	GGAGG	7 bp
TARA_206_MES_92546	2,768	ORF1	+	332	2,650	RdRP	772	MINPKNKHPLLTMFNDEPIRVNN	n/a	-	AGGAGG	5 bp
TARA_132_DCM_33585	3,245	ORF1	+	157	2,556	RdRP	799	MKDFNETQSKNKKQSQSPPEIVV	n/a	-	AGGAGG	7 bp
		ORF2	+	2,540	3,139		199	MNNKHETKENERRKKAGSGQRS	n/a	-	3Base/5BMM	13-15 bp

[1] Reference: Steinegger M *et al.* (2019) *BMC Bioinformatics* **20**: 473. https://doi.org/10.1186/s12859-019-3019-7
