# Supplementary material for: “Paraxenoviridae”, a putative family of globally distributed marine bacteriophages with double-stranded RNA genomes
Source: ISME J. 2025 Jul 4;19(1):wraf139. doi: 10.1093/ismejo/wraf139 (PMC12445693; doi:10.1093/ismejo/wraf139)
Supplement: 20250508_TableS5_wraf139 [file 20250508_tables5_wraf139.pdf]

Table S5. FoldSeek searches against BFVD database queried with the RdRP structural models

Query	Target	Scientific Name	Prob.	Seq. Id.	E-Value	Score	Query Pos.	Target Pos.
GT3	A0A6B9UQ12	Mongoose picobirna-like virus Mongoose/KNA/M17A/2017	1	13.5	3.98E-11	365	116-801 (871)	2-486 (510)
GT3	A0A858HW50	Riboviria sp.	1	12.3	2.51E-11	324	136-742 (871)	7-467 (478)
GT3	A0A8F6QH75	Picobirnaviridae sp. gcode 3	1	12.2	1.31E-10	315	121-837 (871)	8-546 (558)
GT3	A0A8K1XI45	Yellowtail scad partiti-like virus	1	10.4	9.51E-11	282	134-801 (871)	22-537 (539)
GT3	A0A6H0DIB1	Ostrekin virus	1	13.2	2.25E-09	261	179-797 (871)	127-572 (589)
GT3	A0A4P8WE71	Bat picobirnavirus C1	1	13.8	6.12E-08	254	153-687 (871)	10-390 (393)
GT3	A0A8K1U2M8	Riboviria sp.	1	12.4	2.04E-08	246	187-763 (871)	21-422 (433)
GT3	A0A1L3KLN2	Hubei partiti-like virus 57	1	11.1	5.90E-09	245	103-762 (871)	34-483 (504)
GT3	A0A1L3KMD9_2	Wenzhou picorna-like virus 13	1	10.1	1.94E-08	244	173-797 (871)	81-558 (1439)
GT3	A0A8D9PCQ8	Penicillium stoloniferum virus F	1	11.8	1.24E-09	243	62-762 (871)	1-502 (537)
GT4	A0A6B9UQ12	Mongoose picobirna-like virus Mongoose/KNA/M17A/2017	1	13.2	6.13E-15	488	59-717 (748)	18-510 (510)
GT4	A0A858HW50	Riboviria sp.	1	12.7	7.64E-14	378	72-677 (748)	13-476 (478)
GT4	A0A8K1XI45	Yellowtail scad partiti-like virus	1	12	1.11E-13	371	73-708 (748)	22-539 (539)
GT4	A0A1L3KLD8	Hubei partiti-like virus 7	1	14.5	3.21E-12	364	68-700 (748)	30-501 (540)
GT4	A0A1L3KLL0	Hubei partiti-like virus 54	1	13.3	2.92E-12	355	34-696 (748)	10-467 (478)
GT4	A0A8D9PCQ8	Penicillium stoloniferum virus F	1	13.6	7.20E-13	351	44-661 (748)	31-502 (537)
GT4	A0A8F6QH75	Picobirnaviridae sp. gcode 3	1	11.7	6.47E-12	348	47-720 (748)	2-550 (558)
GT4	M9TFL3	Ustilaginoidea virens partitivirus 3	1	11.5	1.50E-11	347	72-722 (748)	42-521 (522)
GT4	A0A7I8CXE8	Aspergillus lentulus partitivirus 1	1	11.7	2.39E-11	339	72-705 (748)	108-578 (582)
GT4	A0A1L3KLN2	Hubei partiti-like virus 57	1	14.1	6.78E-12	332	72-707 (748)	34-502 (504)
GT5	A0A6B9UQ12	Mongoose picobirna-like virus Mongoose/KNA/M17A/2017	1	10.8	6.76E-11	326	75-733 (806)	2-480 (510)
GT5	A0A858HW50	Riboviria sp.	1	10.2	5.47E-09	291	178-702 (806)	80-466 (478)
GT5	A0A1L3KLD8	Hubei partiti-like virus 7	1	10.6	4.76E-09	283	155-728 (806)	71-490 (540)
GT5	A0A1L3KLN2	Hubei partiti-like virus 57	1	9.3	1.72E-08	277	155-695 (806)	79-458 (504)
GT5	A0A7T7K918	Notsystermes virus	1	9.6	1.80E-08	271	47-804 (806)	25-638 (640)
GT5	A0A8D9PCQ8	Penicillium stoloniferum virus F	1	11.7	1.52E-09	269	57-694 (806)	23-519 (537)
GT5	A0A1L3KLL0	Hubei partiti-like virus 54	1	11.2	3.57E-08	268	58-679 (806)	7-423 (478)
GT5	A0A8K1XI45	Yellowtail scad partiti-like virus	1	10	4.41E-10	267	98-806 (806)	22-539 (539)
GT5	A0A023IN43_2	limnipivirus C1	1	9.4	1.14E-08	256	154-728 (806)	695-1126 (1155)
GT5	A0A8F6QH75	Picobirnaviridae sp. gcode 3	1	9	7.53E-09	251	75-761 (806)	2-550 (558)
TARA132	A0A6B9UQ12	Mongoose picobirna-like virus Mongoose/KNA/M17A/2017	1	12.6	2.94E-11	392	57-757 (799)	10-510 (510)
TARA132	A0A8F6QH75	Picobirnaviridae sp. gcode 3	1	11.8	1.86E-11	349	57-774 (799)	2-557 (558)

TARA132	A0A1L3KLN2	Hubei partiti-like virus 57	1	13.4	4.86E-11	342	50-682 (799)	20-455 (504)
TARA132	A0A8D9PCQ8	Penicillium stoloniferum virus F	1	10.4	7.67E-11	337	54-663 (799)	31-467 (537)
TARA132	M9TFL3	Ustilaginoidea virens partitivirus 3	1	12.2	4.15E-10	326	81-673 (799)	42-458 (522)
TARA132	A0A1L3KLL0	Hubei partiti-like virus 54	1	12.4	5.21E-10	319	53-683 (799)	13-430 (478)
TARA132	A0A2P0XJ43	Partitiviridae sp.	1	13.4	7.50E-10	313	98-775 (799)	2-519 (643)
TARA132	A0A1L3KLD8	Hubei partiti-like virus 7	1	13.4	2.75E-10	312	77-745 (799)	30-516 (540)
TARA132	A0A858HW50	Riboviria sp.	1	12.6	8.40E-11	310	81-683 (799)	13-460 (478)
TARA132	A0A6H0DIB1	Ostrekin virus	1	11.7	7.67E-11	303	32-765 (799)	87-586 (589)
