# Supplementary material for: “Paraxenoviridae”, a putative family of globally distributed marine bacteriophages with double-stranded RNA genomes
Source: ISME J. 2025 Jul 4;19(1):wraf139. doi: 10.1093/ismejo/wraf139 (PMC12445693; doi:10.1093/ismejo/wraf139)
Supplement: 20250508_TableS6_wraf139 [file 20250508_tables6_wraf139.pdf]

Table S6. FoldSeek searches against PDB100 database queried with the RdRP structural models

Query	Target	Taxonomy Name (ID)	Prob.	PDB id	E-Value	Score	Query Pos.	Target Pos.
GT3	5i61-assembly1.cif.gz_A Crystal structure of the RNA-dependent RNA polymerase of a human picorbirnavirus	Human picobirnavirus strain Hy005102 (647332)	1	5i61	8.16E-10	293	137-812 (871)	31-523 (523)
GT3	7dfg-assembly1.cif.gz_A Structure of COVID-19 RNA-dependent RNA polymerase bound to favipiravir	Severe acute respiratory syndrome coronavirus 2 (2697049)	1	7dfg	4.42E-08	202	138-823 (871)	398-901 (908)
GT3	5yf5-assembly1.cif.gz_A Crystals structure of Classical swine fever virus NS5B (residues 1-694)	Classical swine fever virus (11096)	1	5yf5	3.81E-07	193	224-748 (871)	167-543 (641)
GT3	4nrt-assembly1.cif.gz_A Human Norovirus polymerase bound to Compound 6 (suramin derivative)	Norwalk-like virus (95340)	1	4nrt	6.03E-07	186	189-742 (871)	48-461 (500)
GT3	1sh0-assembly2.cif.gz_B Crystal Structure of Norwalk Virus Polymerase (Triclinic)	Norwalk virus (11983)	1	1sh0	6.61E-07	180	222-754 (871)	80-471 (503)
GT3	3nky-assembly1.cif.gz_A Structure of a mutant P44S of Foot-and-mouth disease Virus RNA-dependent RNA polymerase	Foot and mouth disease virus C (12116)	1	3nky	8.32E-07	170	212-772 (871)	59-470 (476)
GT3	8g6r-assembly1.cif.gz_A Porcine epidemic diarrhea virus core polymerase complex	Porcine epidemic diarrhea virus (28295)	1	8g6r	9.12E-07	183	189-835 (871)	427-895 (899)
GT3	2cjq-assembly1.cif.gz_A Bovine viral diarrhea virus CP7-R12 RNA-dependent RNA polymerase	Bovine viral diarrhea virus 1 (11099)	1	2cjq	9.54E-07	186	219-752 (871)	78-448 (546)
GT3	2wk4-assembly1.cif.gz_A Dimeric structure of D347G D348G mutant of the sapporovirus RNA dependent RNA polymerase	Sapporo virus (95342)	1	2wk4	1.20E-06	191	193-833 (871)	53-487 (499)
GT3	4wzm-assembly1.cif.gz_A Mutant K18E of RNA dependent RNA polymerase from Foot-and-Mouth Disease Virus complexed with RNA	Foot-and-mouth disease virus (strain C1-Santa Pau) (12120)	1	4wzm	1.51E-06	173	191-766 (871)	49-464 (475)
GT4	5i61-assembly1.cif.gz_A Crystal structure of the RNA-dependent RNA polymerase of a human picorbirnavirus	Human picobirnavirus strain Hy005102 (647332)	1	5i61	1.53E-13	428	60-717 (748)	20-522 (523)
GT4	7dfg-assembly1.cif.gz_A Structure of COVID-19 RNA-dependent RNA polymerase bound to favipiravir	Severe acute respiratory syndrome coronavirus 2 (2697049)	1	7dfg	7.56E-10	260	66-694 (748)	391-902 (908)
GT4	4nlp-assembly1.cif.gz_A Poliovirus Polymerase - C290V Loop Mutant	Human poliovirus 1 Mahoney (12081)	1	4nlp	7.46E-09	268	152-691 (748)	72-457 (458)
GT4	4zp7-assembly1.cif.gz_A Coxsackievirus B3 Polymerase - F364V mutant	Coxsackievirus B3 (12072)	1	4zp7	8.58E-09	256	152-707 (748)	72-453 (456)
GT4	5ziu-assembly1.cif.gz_A Crystal structure of human Entervirus D68 RdRp	enterovirus D68 (42789)	1	5ziu	9.42E-09	277	142-661 (748)	57-433 (457)
GT4	3n6n-assembly1.cif.gz_A crystal structure of EV71 RdRp in complex with Br-UTP	Enterovirus A71 (39054)	1	3n6n	1.08E-08	232	146-663 (748)	66-461 (462)
GT4	3cdw-assembly1.cif.gz_A Crystal structure of coxsackievirus B3 RNA-dependent RNA polymerase (3Dpol) in complex with protein primer	Coxsackievirus B3 (strain Nancy) (103903)	1	3cdw	1.19E-08	258	99-659 (748)	44-436 (468)
GT4	2ckw-assembly1.cif.gz_A The 2.3 A resolution structure of the Sapporo virus RNA dependant RNA polymerase.	Sapporo virus (95342)	1	2ckw	1.50E-08	255	123-664 (748)	56-463 (487)
GT4	4iqx-assembly1.cif.gz_A Mutant P44S P169S M296I of Foot-and-mouth disease Virus RNA-dependent RNA polymerase	Foot and mouth disease virus C (12116)	1	4iqx	1.58E-08	233	128-695 (748)	52-473 (476)
GT4	1xr7-assembly2.cif.gz_B Crystal structure of RNA-dependent RNA Polymerase 3D from human rhinovirus serotype 16	rhinovirus A16 (31708)	1	1xr7	2.09E-08	262	152-677 (748)	72-450 (460)
GT5	5i62-assembly1.cif.gz_A Crystal structure of the insertion loop deletion mutant of the RNA-dependent RNA polymerase of a human picorbir	Human picobirnavirus strain Hy005102 (647332)	1	5i62	1.58E-09	294	84-707 (806)	19-475 (507)
GT5	7c2k-assembly1.cif.gz_A COVID-19 RNA-dependent RNA polymerase pre-translocated catalytic complex	Severe acute respiratory syndrome coronavirus 2 (2697049)	1	7c2k	9.41E-09	236	91-759 (806)	397-922 (927)
GT5	2wk4-assembly1.cif.gz_A Dimeric structure of D347G D348G mutant of the sapporovirus RNA dependent RNA polymerase	Sapporo virus (95342)	1	2wk4	2.42E-07	220	144-724 (806)	46-485 (499)
GT5	6gp9-assembly1.cif.gz_A Structural studies of hepatitis C virus non-structural protein-5b of genotype 4a	Hepacivirus hominis (11103)	1	6gp9	6.63E-07	179	175-758 (806)	58-533 (553)
GT5	4qpx-assembly1.cif.gz_A NV polymerase post-incorporation-like complex	Norwalk virus (11983)	1	4qpx	6.94E-07	197	148-714 (806)	52-488 (495)
GT5	6nur-assembly1.cif.gz_A SARS-Coronavirus NSP12 bound to NSP7 and NSP8 co-factors	Severe acute respiratory syndrome-related coronavirus (227859)	1	6nur	7.97E-07	196	121-695 (806)	301-767 (793)
GT5	8urb-assembly1.cif.gz_A Porcine epidemic diarrhea virus complete core polymerase complex	Porcine epidemic diarrhea virus (28295)	1	8urb	9.56E-07	176	91-759 (806)	390-917 (921)
GT5	3qgd-assembly2.cif.gz_B Crystal structure of the hepatitis C virus NS5B RNA-dependent RNA polymerase complex with (2E)-3-(4-{[(1-{[(	Hepatitis C virus subtype 1b (31647)	1	3qgd	2.18E-06	165	167-752 (806)	15-500 (515)
GT5	4dru-assembly2.cif.gz_B HCV NS5B in complex with macrocyclic INDOLE INHIBITOR	Hepatitis C virus isolate HC-J4 (420174)	1	4dru	3.00E-06	163	175-752 (806)	58-523 (559)
GT5	4wzq-assembly1.cif.gz_A Mutant K20E of RNA dependent RNA polymerase 3D from Foot-and-Mouth disease Virus complexed with RNA	Foot-and-mouth disease virus (12110)	1	4wzq	3.61E-06	161	161-699 (806)	45-466 (476)
TARA132	5i61-assembly1.cif.gz_A Crystal structure of the RNA-dependent RNA polymerase of a human picorbirnavirus	Human picobirnavirus strain Hy005102 (647332)	1	5i61	1.73E-10	357	68-757 (799)	19-522 (523)
TARA132	7dfg-assembly1.cif.gz_A Structure of COVID-19 RNA-dependent RNA polymerase bound to favipiravir	Severe acute respiratory syndrome coronavirus 2 (2697049)	1	7dfg	5.31E-09	229	30-772 (799)	361-908 (908)
TARA132	8urb-assembly1.cif.gz_A Porcine epidemic diarrhea virus complete core polymerase complex	Porcine epidemic diarrhea virus (28295)	1	8urb	7.15E-08	213	32-690 (799)	362-884 (921)
TARA132	2ckw-assembly1.cif.gz_A The 2.3 A resolution structure of the Sapporo virus RNA dependant RNA polymerase.	Sapporo virus (95342)	1	2ckw	9.84E-08	241	133-726 (799)	56-465 (487)
TARA132	6nur-assembly1.cif.gz_A SARS-Coronavirus NSP12 bound to NSP7 and NSP8 co-factors	Severe acute respiratory syndrome-related coronavirus (227859)	1	6nur	2.24E-07	204	75-682 (799)	281-761 (793)
TARA132	5xe0-assembly1.cif.gz_A Crystal structure of EV-D68-3Dpol in complex with GTP	enterovirus D68 (42789)	1	5xe0	4.86E-07	217	163-669 (799)	69-418 (457)
TARA132	3n6n-assembly1.cif.gz_A crystal structure of EV71 RdRp in complex with Br-UTP	Enterovirus A71 (39054)	1	3n6n	6.11E-07	201	109-685 (799)	44-435 (462)
TARA132	4iqx-assembly1.cif.gz_A Mutant P44S P169S M296I of Foot-and-mouth disease Virus RNA-dependent RNA polymerase	Foot and mouth disease virus C (12116)	1	4iqx	9.21E-07	197	151-725 (799)	58-471 (476)
TARA132	6gp9-assembly1.cif.gz_A Structural studies of hepatitis C virus non-structural protein-5b of genotype 4a	Hepacivirus hominis (11103)	1	6gp9	1.01E-06	166	110-786 (799)	22-553 (553)
TARA132	3jb7-assembly1.cif.gz_A In situ structures of the segmented genome and RNA polymerase complex inside a dsRNA virus	Bombyx mori cypovirus 1 (110829)	1	3jb7	1.06E-06	140	64-683 (799)	249-776 (1198)
