# Supplementary material for: “Paraxenoviridae”, a putative family of globally distributed marine bacteriophages with double-stranded RNA genomes
Source: ISME J. 2025 Jul 4;19(1):wraf139. doi: 10.1093/ismejo/wraf139 (PMC12445693; doi:10.1093/ismejo/wraf139)
Supplement: 20250702_Paraxeno_ms_revision2_Suppl_fig_legends_wraf139 [file 20250702_paraxeno_ms_revision2_suppl_fig_legends_wraf139.pdf]

Supplementary figure legends

Fig. S1. RdRP-encoding genome (RNA1) segments of paraxenoviruses from pelagic dsRNA viromes. ORFs encoding homologous proteins are shown as arrows with identical colors. Yellow circles represent predicted SD RBS motifs. Asterisks denote putative genes encoding predicted transmembrane domain (TMD)-containing proteins.

Fig. S2. Non-RdRP-encoding genome (RNA2) segments of paraxenoviruses from pelagic dsRNA viromes. ORFs encoding homologous proteins are shown as arrows with identical colors. Yellow circles represent predicted SD RBS motifs.

Fig. S3. Previously reported TARA virus contigs encoding '*Ca.* Paraxenoviricota'-associated RdRP proteins [11]. Open reading frames encoding homologous RdRP proteins are shown as arrows with orange color. Yellow circles represent predicted SD RBS motifs.

Fig. S4. An internal phylogeny within the clade "paraxenoviruses", close-up from Figure 3. The phylogenetic relationships among GT1–5 RdRPs, TARA RdRPs, and their close relatives (ID numbers with the prefix 'Ga') within the paraxenoviral group are shown.

Fig. S5. Structural models of paraxenovirus RdRPs. For each of RdRPs from TARA_132, GT4, GT3, and GT5 paraxenoviruses, ColabFold prediction was performed using five alphafold2_ptm_models and five seeds (000 to 004). The resulting 25 models were displayed, superimposed by the Matchmaker tool in ChimeraX, and colored by the pLDDT confidence score. The left column shows a representative, AMBER-relaxed model from seed_000 for each RdRP, as shown in Fig. 4A. The right column shows the 25 superimposed models for each RdRP. Except for a few low confident regions (yellow to orange), most of the domains are predicted consistently and invariably among the 25 models. The confidence scores of all of the RdRP models are summarized in Table S2.

Fig. S6. Structural models of the paraxenovirus capsid proteins. (A) Capsid protein of GT2. (B) Capsid protein of GT4. The domain organizations are indicated. The models are colored based on the confidence values of the predicted local distance difference test (pLDDT), with the scale provided at the bottom of the figure. (C) Model of GT2 capsid dimer. Model is colored by the domain organization. (D) Predicted aligned error (PAE) plot of GT2 capsid dimer. The domain organizations are indicated.

Fig. S7. Structural model of potential capsid protein ORF1 GT5_RNA2. The predicted structure of ORF1 GT5_RNA2 (yellow) is overlapped with the model of GT2 capsid protein (blue). Structural features shared by two models are highlighted with less transparent color. The domain organizations are indicated.
